# Oleanolic Acid-Loaded Cyclodextrin Metal–Organic Framework Ameliorates NAFLD via Modulation of Gut Microbiota and Liver Metabolism

**DOI:** 10.3390/molecules31203663

**Published:** 2026-10-13

**Authors:** Hua Zhu, Minmin Tang, Meng Li, Zhenhua Zhang, Liqin Kang, Qiaona Wang, Renlei Wang, Chun Hua, Huiming Zhou, Wenzhi Zhou, Yinliang Wu, Zhenjiong Wang

**Affiliations:** 1 Key Laboratory of Innovative Applications of Bioresources and Functional Molecules of Jiangsu Province, College of Life Science and Chemistry, Jiangsu Second Normal University, Nanjing 211200, China; 2 Key Laboratory of Food Contact Materials Safety, State Administration for Market Regulation, Jiangsu Product Quality Testing & Inspection Institute, Nanjing 210007, China; 3 Collaborative Innovation Center for Modern Grain Circulation and Safety, College of Food Science and Engineering, Nanjing University of Finance and Economics, Nanjing 210023, China; 4 School of Ecology and Applied Meteorology, Nanjing University of Information Science & Technology, Nanjing 210044, China; 5 School of Food Science, Nanjing Xiaozhuang University, Nanjing 211171, China; 6 Jiangsu Sanshu Biotechnology Go., Ltd., No. 259 Shibei Gaoxin Road, Chongchuan District, Nantong 226001, China

**Keywords:** oleanolic acid, metal–organic framework, high-fat diet, cyclodextrin, liver metabolomics, gut microbiota

## Abstract

Oleanolic acid (OA) possesses hepatoprotective properties, but its clinical application is hampered by poor aqueous solubility. This study aimed to enhance OA solubility and investigate its therapeutic mechanism for NAFLD by encapsulating it in a γ-cyclodextrin metal–organic framework (OA@CDMOF). The OA@CDMOF complex was synthesized and characterized. Its effects on a high-fat diet (HFD)-induced NAFLD mouse model (MOD) were evaluated by combined liver metabolomics and 16S rRNA amplicon sequencing analyses. When encapsulated into CDMOF to yield an OA@CDMOF complex, the solubility of OA significantly improved. OA@CDMOF administration ameliorated HFD-induced metabolic alterations and liver injury. It modulated the gut microbiota composition, notably enriching the phylum Firmicutes while decreasing the abundance of Bacteroidota. Liver metabolomic analysis revealed that OA@CDMOF was associated with changes in metabolites related to glycerophospholipid, purine, glutathione, and vitamin B6 metabolism. Furthermore, it was associated with changes in metabolites related to lipolysis in adipocytes, pentose and glucuronate interconversions, and key signaling pathways, including AMPK, FoxO, and cGMP-PKG. Importantly, OA@CDMOF showed protective effects comparable to those of free OA, with no statistically significant difference between the two groups. This study elucidates the mechanism by which OA@CDMOF alleviates NAFLD through modulation of the gut microbiome and host metabolism. Our findings provide novel insights into the hepatoprotective role of OA@CDMOF and suggest a promising nanostrategy for NAFLD intervention.

## 1. Introduction

Nonalcoholic fatty liver disease (NAFLD) is associated with increased hepatic fat accumulation or steatosis, which can potentially lead to nonalcoholic steatohepatitis (NASH), fibrosis, and ultimately cirrhosis [1]. Approximately 32.4% of the global population suffers from NAFLD [2]. Despite extensive research into its pathogenesis, the precise mechanisms underlying NAFLD remain unclear, thus impeding the development of appropriate pharmaceutical treatments. Indeed, pharmacological treatment options for NAFLD remain limited. While resmetirom (Rezdiffra) was approved by the FDA in March 2024 for the treatment of NASH with moderate to advanced fibrosis, no FDA-approved medication currently exists for early-stage NAFLD without significant fibrosis, and treatment strategies generally continue to focus on dietary changes and lifestyle adjustments.

NAFLD has a complex pathophysiology, described as a “multiple parallel-hit hypothesis” involving changes in cytokine levels, increased free fatty acids and insulin resistance associated with obesity and subsequent NAFLD [3]. The liver is the central organ that regulates lipid metabolism, specifically fatty acid metabolism, which is controlled by intricate interactions between receptors, hormones, and transcription factors [4]. Disruption of one or more of these pathways may increase liver fat accumulation and impair fatty acid oxidation. Such an imbalance not only prevents lipid levels from being normalized but also may accelerate damage to cells and exacerbate NAFLD by promoting oxidative stress. Increased apoptosis resulting from hepatic steatosis may lead to broader metabolic disorders that affect multiple organs [5]. In addition, the gut plays a significant role in NAFLD progression through various mechanisms [6]. In this context, research has indicated that gut microbiota imbalances, compromised intestinal barriers and immune responses are interconnected in the development of liver diseases [7]. In NAFLD, disruptions in the gut microbiota and increased intestinal permeability can lead to liver damage caused by microbial metabolites, potentially causing endotoxemia and altering gut–liver axis communication [8]. The portal vein is responsible for the transportation of microbial products to the liver, while bile and antibodies travel back to the gut through hepatic pathways. A healthy gut–liver axis requires proper liver function, a balanced gut microbiota and an intact intestinal barrier. However, compromise of the intestinal barrier leading to the transport of bacteria and metabolites to the liver triggers immune and inflammatory responses that contribute to NAFLD progression [9]. Hence, preserving gut health has become important for predicting NAFLD severity and offers new potential avenues for treatment.

Oleanolic acid (OA), one of the most well-researched pentacyclic triterpenoids, has demonstrated numerous benefits in managing metabolic diseases through different mechanisms [10]. For instance, OA was shown to enhance gustatory fat perception through increased levels of taste bud receptors in mice, thus helping to reduce diet-induced obesity [11]. Furthermore, OA can modulate the liver levels of circadian genes through the suppression of lipid accumulation by apolipoprotein A1 [12]. Moreover, in addition to its metabolic benefits, OA has been shown to reinforce the intestinal barrier. In particular, it mitigated intestinal inflammatory responses triggered by *Salmonella typhimurium* in mice by inhibiting the TLR4/NF-κB/MAPK axis [13]. However, the low oral bioavailability, chemical instability and poor aqueous solubility of OA present challenges for further clinical translation [14,15]. The development of effective delivery approaches or formulations is needed to improve its drug-like properties.

Improving aqueous solubility, solution stability, and bioavailability is the key to addressing the poor drug release of herbal monomers after delivery to the target organ. Cyclodextrins (CDs) are biocompatible cyclic oligosaccharides with a hydrophobic core and hydrophilic exterior, rendering them water soluble. They have been commonly used for encapsulating volatile active ingredients because they enable controlled release through supramolecular interactions. In particular, γ-CD-based metal–organic frameworks (CDMOFs), formed from alkali metal cations (such as K^+^) and γ-CDs, have attracted significant attention because of their porous structure, which allows the encapsulation of specific molecules [16,17]. In CDMOF, K^+^ ions coordinate with the hydroxyl groups of adjacent γ-CD molecules, bridging them to form a three-dimensional porous framework with well-defined channels and cavities, in which each cubic unit built from six γ-CD molecules encloses a spherical nanocage approximately 1.7 nm in diameter [16,17], providing sufficient space for accommodating larger guest molecules such as OA. In this context, previous research has shown that CDMOF is highly effective at encapsulating various hydrophobic bioactive molecules, such as folic acid [18], quercetin [19], isosteviol [20] and curcumin [21], with excellent loading efficiency. Moreover, encapsulating those bioactive compounds in CDMOF significantly improves their solubility and bioavailability [22]. However, to date, there has been no systematic investigation of the potential of CDMOF as a solubilizer or encapsulation agent for OA. On the basis of the above, it is hypothesized that encapsulating OA within the porous structure of CDMOF may increase its dissolution, stability and in vivo performance.

This study investigated the feasibility of using CDMOF to encapsulate OA, and the effects of OA@CDMOF formulations on inflammation, oxidative stress, disrupted lipid metabolism and pathological changes associated with NAFLD were evaluated. Additionally, liver metabolomics was performed to assess the global metabolic effects of dietary OA@CDMOF on liver function, including the underlying mechanisms. Finally, the changes in the gut microbiota following OA@CDMOF administration were explored. These results may help to elucidate the active components of OA@CDMOF that have therapeutic potential for NAFLD treatment while clarifying its associations with the gut and liver. This study highlights the need to explore the applicability of CDMOF for oral administration, with the goal of increasing the bioavailability and efficacy of OA. The establishment of an optimized CDMOF oral delivery platform has the potential to fully harness the therapeutic efficacy of OA for combating NAFLD.

## 2. Results and Discussion

### 2.1. Preparation and Analysis of OA@CDMOF

In our study, the OA content in OA@CDMOF was assessed by HPLC analysis of the samples in water. The OA content in CDMOF reached 11.38 ± 2.65 mg/g (i.e., 1.138% *w*/*w*), and the encapsulation efficiency of OA was 17.97 ± 1.13%. Compared with previously reported CDMOF formulations, such as those with a curcumin loading of approximately 16% *w*/*w* [21] and an isosteviol loading of approximately 8.5% *w*/*w* [20], the OA loading of 1.138% *w*/*w* obtained in this study was relatively low, which may be attributed to the relatively large molecular size of OA and the specific loading conditions used. In addition, the relatively large variability in drug content (11.38 ± 2.65 mg/g, coefficient of variation ≈ 23%) may raise concerns about batch reproducibility, potentially reflecting heterogeneity in the loading process, incomplete dispersion, and low overall drug content, amplifying measurement errors. Future work should optimize the loading protocol to improve reproducibility.

**Figure 1:**
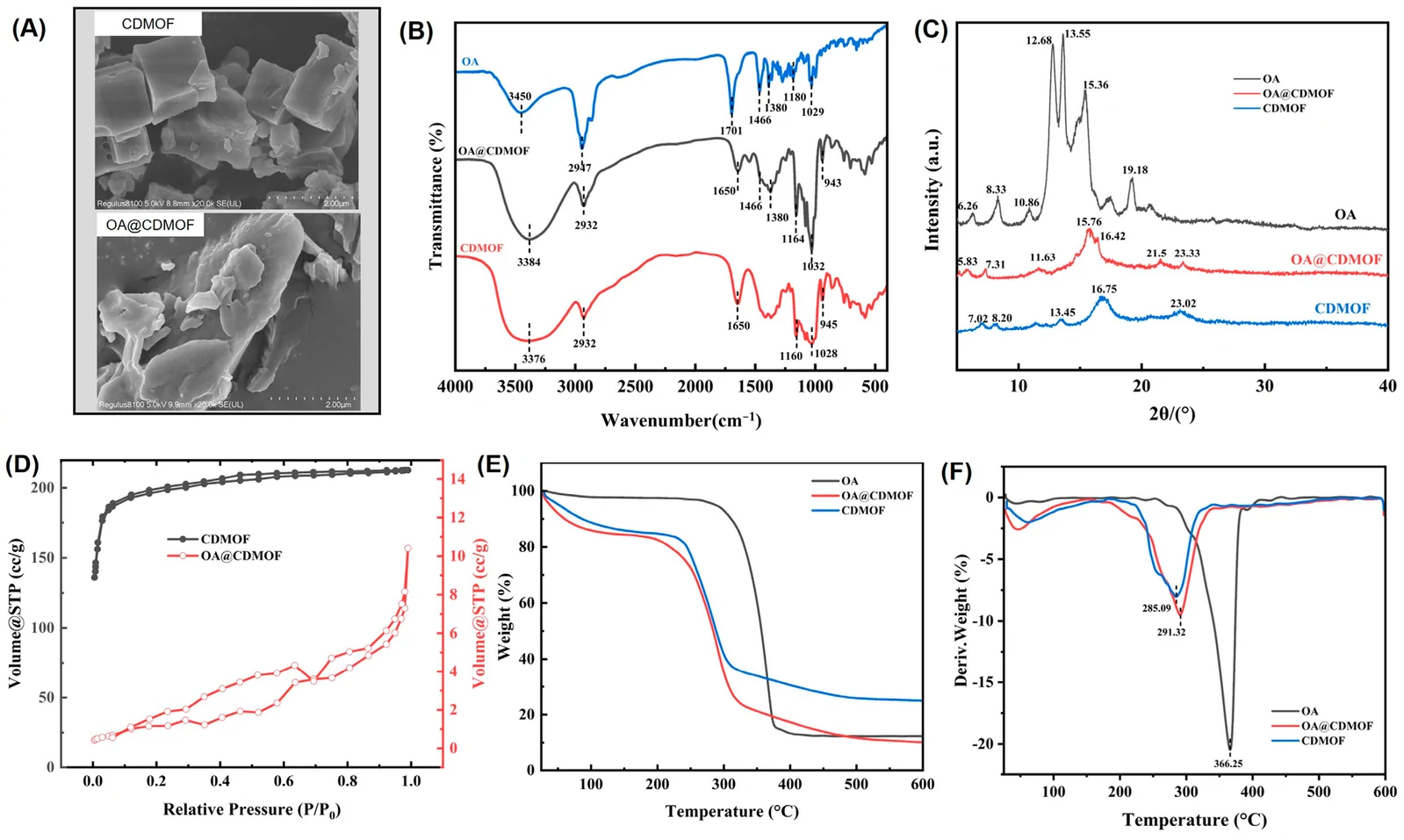
Sample characterization based on (**A**) SEM images of CDMOF and OA@CDMOF; (**B**) FTIR spectra of OA, OA@CDMOF and CDMOF; (**C**) XRD patterns and (**D**) N_2_ adsorption isotherms; TGA (**E**) and DTG (**F**) curves of OA, OA@CDMOF and CDMOF.

On the basis of the SEM images (Figure 1A), CDMOF was found to exhibit cubic morphologies, as reported in the literature [23]. However, the morphologies of OA@CDMOF presented disintegrated structures, suggesting morphological changes upon OA loading. These results indicate possible OA incorporation into CDMOF, although SEM alone cannot confirm internal pore localization.

The FTIR spectrum of OA (Figure 1B) exhibited OH stretching vibrations at 3450 cm^−1^, whereas the stretching vibrations of methylene and carbonyl moieties (C=O) of carboxyls (COOH) were indicated by peaks at 2947 cm^−1^ and 1701 cm^−1^, respectively [24]. In the case of OA@CDMOF, the C=O stretching peak at approximately 1701 cm^−1^ was not clearly distinguishable, which may be attributed to the low OA content (1.138% *w*/*w*) and spectral overlap with the CDMOF bands, rather than indicating a chemical reaction. The OH stretching region in OA@CDMOF showed a broad band centered at approximately 3384 cm^−1^, reflecting contributions from both OA and CDMOF. In addition, the peaks at 1466 and 1380 cm^−1^ represented C–H bending, and there were no obvious differences between OA and OA@CDMOF in this region.

The synthesis of CDMOF was confirmed on the basis of similarities between its diffraction peaks (Figure 1C) and those reported previously [25]. Pure OA exhibited sharp and intense peaks at 2θ = 6.26°, 8.33°, 10.86°, 12.68°, 13.55°, 15.36° and 19.18°, indicating that pure OA was a crystalline material. However, the XRD pattern of OA@CDMOF did not show OA-related peaks, suggesting that OA was incorporated into the CDMOF framework. The retention of the CDMOF diffraction pattern indicates that the crystalline structure of the framework is preserved after OA loading.

After OA loading, the BET surface area of OA@CDMOF (4.725 ± 0.322 m^2^/g) was markedly lower than that of CDMOF (794.609 ± 17.6 m^2^/g). This dramatic reduction (>99%) indicates significant structural changes after OA loading, which may involve pore blocking, framework collapse, or a combination of factors (Figure 1D).

CDMOF and OA@CDMOF exhibited thermal stability between 30 °C and 200 °C (Figure 1E,F). The derivative thermogravimetry (DTG) curves (Figure 1F) indicated a peak representing the maximum weight loss temperature (Tp). Compared with pure OA (366.25 °C), OA@CDMOF (291.32 °C) had a lower Tp. Taken together with the FTIR, PXRD, and BET results, these data support the incorporation of OA into the CDMOF framework.

### 2.2. Solubility Analyses

This study also explored how OA solubility was altered following encapsulation in CDMOF. After OA@CDMOF was synthesized, the solubility changed from 0.0145 mg/L to 1.4333 mg/L, with this change representing an approximately 100-fold increase. This difference could be attributed to OA encapsulation within the CDMOF framework [26]. Such solubility improvement may facilitate the dissolution and absorption of OA in the gastrointestinal tract. Accordingly, we further evaluated the intervention effect of OA@CDMOF in NAFLD models. However, OA solubility in simulated gastric and intestinal fluids was not characterized in this work, which represents a limitation to be addressed in future formulation optimization and pharmacokinetic studies.

### 2.3. OA@CDMOF Reduces Body Weight and Hepatic Fat Accumulation in HFD-Induced NAFLD Mice

**Figure 2:**
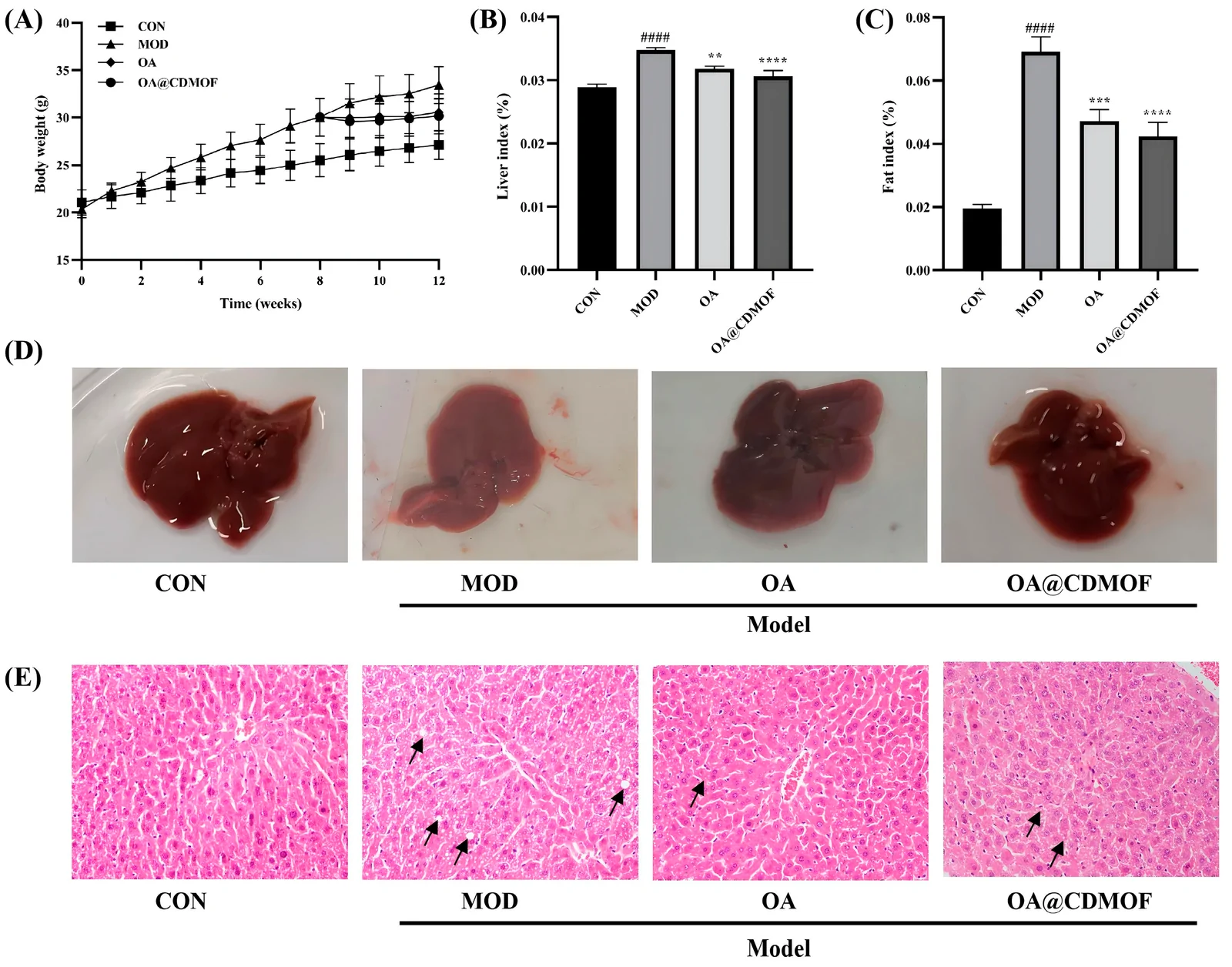
OA@CDMOF reduces hepatic lipid accumulation. (**A**) Changes in weight as determined every 7 days; (**B**) liver index; (**C**) fat index; (**D**) liver tissue photos; (**E**) images of H&E-stained liver tissues (magnification 200×). Different letters on the same parameter indicate significant differences. Significantly different values are indicated by different superscripts (*p* < 0.05). The data are presented as the mean ± SEM (*n* = 8). ^####^ *p* < 0.0001 compared with the CON group; ** *p* < 0.01, *** *p* < 0.001, **** *p* < 0.0001 compared with the MOD group. Pairwise comparisons between OA and OA@CDMOF were performed using Tukey’s post hoc test; no statistically significant difference was observed (*p* > 0.05, n.s.). Arrows in (**E**) indicate lipid droplets.

To investigate the therapeutic efficacy of OA@CDMOF on NAFLD, C57BL/6J mice were fed a high-fat diet (HFD) with or without OA@CDMOF intervention. As shown in Figure 2A, compared with the OA and OA@CDMOF groups, the MOD group exhibited significantly greater body weights starting at week 8 of feeding. By 12 weeks, the mean body weight in the MOD group had increased by 64.35% (33.45 g), whereas for those on a normal diet, only a 28.72% increase was observed (27.11 g). Supplementation with 5 mg/kg OA or OA@CDMOF, starting at week 8, decreased body weight gain. No statistically significant difference was observed between the OA and OA@CDMOF groups (Tukey’s post hoc test, *p* > 0.05; Figure 2A). Consistent with the body weight changes, compared with the CON group, the MOD group exhibited significantly greater liver weights and liver indices (*p* < 0.001), whereas OA@CDMOF treatment significantly reversed these alterations (Figure 2B,C). Similarly, the overall morphology and enlarged volume of livers in MOD mice, along with their pale yellow appearance, were substantially alleviated by OA@CDMOF intervention (Figure 2D). H&E staining revealed ballooning degeneration and excessive lipid droplets in the livers of NAFLD mice fed an HFD (Figure 2D), indicating HFD-induced hepatic steatosis. Interestingly, liver sections from the OA and OA@CDMOF groups exhibited very mild hepatic steatosis, particularly in the OA@CDMOF group. Collectively, these findings suggest that OA@CDMOF effectively attenuates hepatic steatosis in NAFLD mice fed a high-fat diet.

### 2.4. OA@CDMOF Improves Serum Biochemical Parameters in HFD-Induced NAFLD Mice

**Figure 3:**
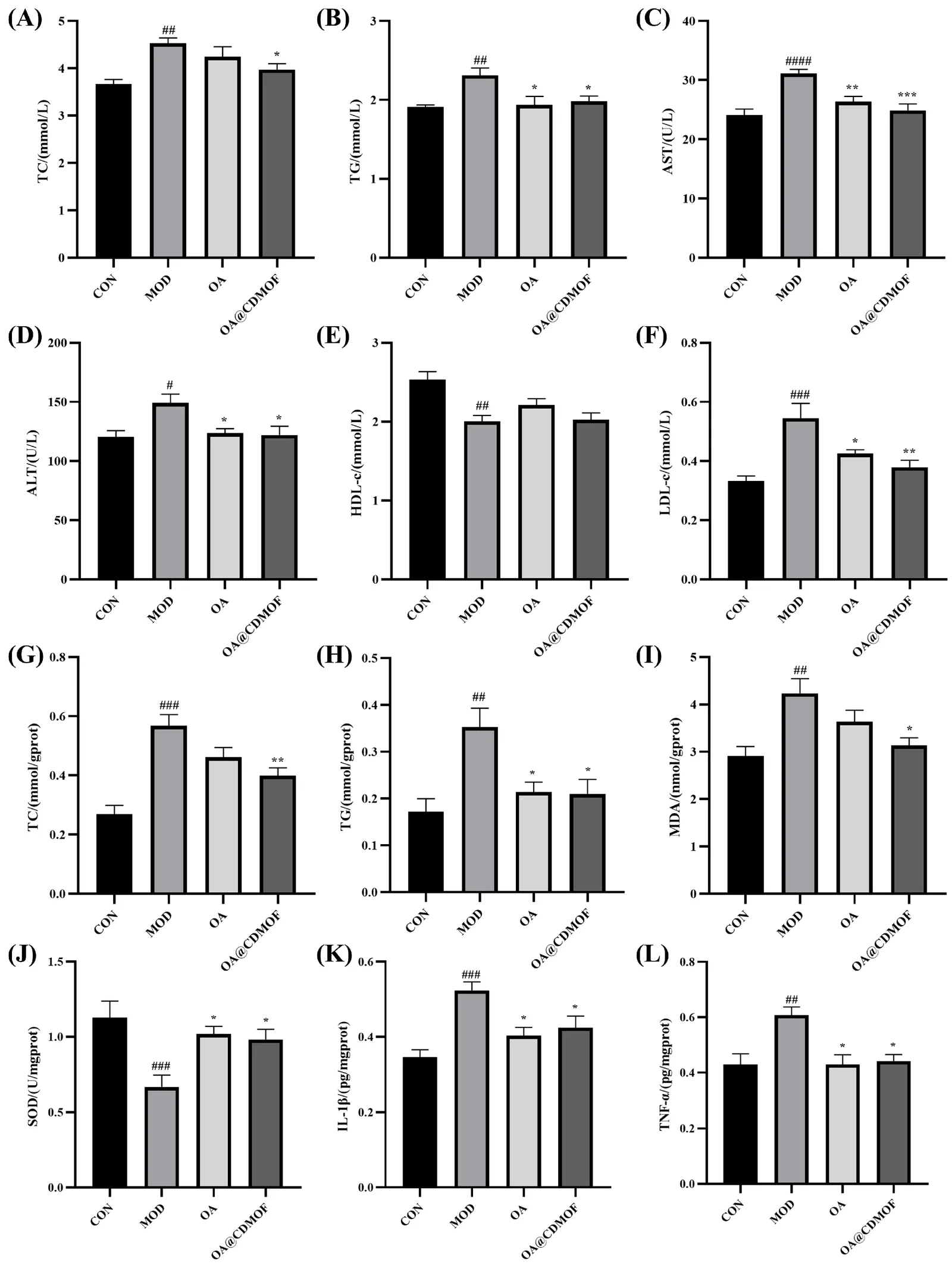
OA@CDMOF reduced NAFLD in mice. (**A**–**F**) Serum TG, TC, AST, ALT, HDL-c, and LDL-c levels. (**G**–**L**) Hepatic TC, TG, and MDA levels; SOD activity; and IL-1β and TNF-α levels. The results are presented as the mean ± SEM (*n* = 8). ^#^ *p* < 0.05, ^##^ *p* < 0.01, ^###^ *p* < 0.001, ^####^ *p* < 0.0001 in comparison with the CON group; * *p* < 0.05, ** *p* < 0.01, *** *p* < 0.001 in comparison with the MOD group. Pairwise comparisons between OA and OA@CDMOF were performed using Tukey’s post hoc test; no statistically significant difference was observed (*p* > 0.05, n.s.).

To evaluate the effects of OA@CDMOF on lipid metabolism and liver injury in high-fat diet (HFD)-induced NAFLD mice, key serum biochemical parameters were measured. As shown in Figure 3A,B, both OA and OA@CDMOF treatment significantly reduced serum total cholesterol (TC) and triglyceride (TG) levels, which were elevated in the MOD group, with TC levels close to those in the CON group. Compared with MOD, both OA@CDMOF and free OA significantly improved serum lipid profiles (Figure 3A–F). The effects of OA@CDMOF were comparable to those of free OA, and no statistically significant difference was found between the two groups (Tukey’s post hoc test, *p* > 0.05).

Liver function was assessed by measuring serum aspartate aminotransferase (AST) and alanine aminotransferase (ALT) levels. Compared with the CON group, the MOD group presented notable increases in the serum levels of both enzymes, whereas OA@CDMOF treatment effectively decreased the serum levels of both enzymes (Figure 3C,D). Elevated ALT and AST levels generally indicate hepatocyte membrane damage and the leakage of enzymes into the circulation, suggesting that HFD intake induces liver injury [27].

In addition, high-density lipoprotein cholesterol (HDL-c) levels remained unchanged across groups (Figure 3E). In contrast, low-density lipoprotein cholesterol (LDL-c) was significantly increased in the MOD group compared with the CON group, and OA@CDMOF effectively reduced its level (Figure 3F). Since LDL-c is the primary cholesterol carrier secreted by the liver, its elevation is closely associated with obesity and metabolic dysregulation [28].

The hepatic TC and TG contents were also determined. Consistent with the serum data, HFD feeding increased hepatic TC and TG levels, whereas both OA and OA@CDMOF decreased their accumulation in the liver (Figure 3G,H). Increased hepatocyte membrane permeability under HFD challenge likely contributes to the release of lipids into the bloodstream [29].

Overall, these results demonstrate that OA@CDMOF effectively ameliorates HFD-induced dyslipidemia and liver injury, as evidenced by the reduction in serum TC, TG, LDL-c, ALT, and AST levels, as well as hepatic TC and TG levels. The bioactive components of OA@CDMOF or their metabolites may exert protective effects. The effects of OA@CDMOF were comparable to those of free OA, and no statistically significant difference was found between the two groups.

### 2.5. OA@CDMOF Alleviates Hepatic Oxidative Stress and Inflammation in HFD-Induced NAFLD Mice

Our findings demonstrate that OA@CDMOF significantly alleviates HFD-induced hepatic oxidative stress and inflammation, two interconnected pathological processes in NAFLD progression. The elevated levels of malondialdehyde (MDA), a key lipid peroxidation product, in the MOD group indicated substantial oxidative damage [30]. Treatment with OA@CDMOF effectively reduced the hepatic MDA content (Figure 3I), suggesting that OA@CDMOF can mitigate lipid peroxidation.

Concurrently, the HFD-induced suppression of the activity of the critical antioxidant enzyme superoxide dismutase (SOD) was reversed by both OA and OA@CDMOF treatments, with no statistically significant difference between the two groups (Figure 3J). The restoration of SOD activity is crucial for counteracting oxidative stress, as this enzyme constitutes a primary cellular defense mechanism by catalyzing the dismutation of superoxide radicals [30,31]. This coordinated reduction in the level of MDA and the restoration of the activity of SOD underscore the efficacy of OA@CDMOF in rebalancing the hepatic redox state.

Furthermore, OA@CDMOF exhibited marked anti-inflammatory effects, as evidenced by the significant attenuation of HFD-induced increases in the proinflammatory cytokines IL-1β and TNF-α (Figure 3K,L). Inflammation is a well-established driver of NAFLD progression, which is consistent with the “multiple-hit” hypothesis [32]. The interplay between oxidative stress and inflammation forms a vicious cycle in NAFLD: lipid overload promotes ROS generation, which in turn activates inflammatory cascades, leading to further hepatic injury and steatosis progression [33,34].

The comparable therapeutic performance of OA@CDMOF and free OA can be attributed to the enhanced solubility afforded by the CDMOF delivery platform. By improving the solubility of OA, the OA@CDMOF complex may ensure more efficient hepatic delivery of the active compound, thereby contributing to the disruption of the critical pathways of oxidative stress and inflammation that underlie NAFLD pathogenesis [35]. While the enhanced aqueous solubility of OA@CDMOF suggests the potential for improved bioavailability, we acknowledge that no pharmacokinetic or tissue distribution studies were conducted in the present work. Improved solubility is necessary but not sufficient for enhanced bioavailability; other factors, including intestinal permeability, first-pass metabolism, and efflux transporter interactions, also play critical roles. Therefore, the suggestion that OA@CDMOF achieves superior efficacy through enhanced bioavailability should be considered a hypothesis that requires confirmation through dedicated pharmacokinetic studies in future work.

In conclusion, the effects of OA@CDMOF were comparable to those of free OA, with no statistically significant difference between the two groups. To elucidate the underlying mechanism of action, combined metabolomics and 16S rRNA amplicon sequencing analyses were employed. This strategy provided systems-level insight into how OA@CDMOF ameliorates NAFLD by modulating host metabolism and the gut microbiota.

### 2.6. Effects of OA@CDMOF Supplementation on Liver Metabolomes

**Figure 4:**
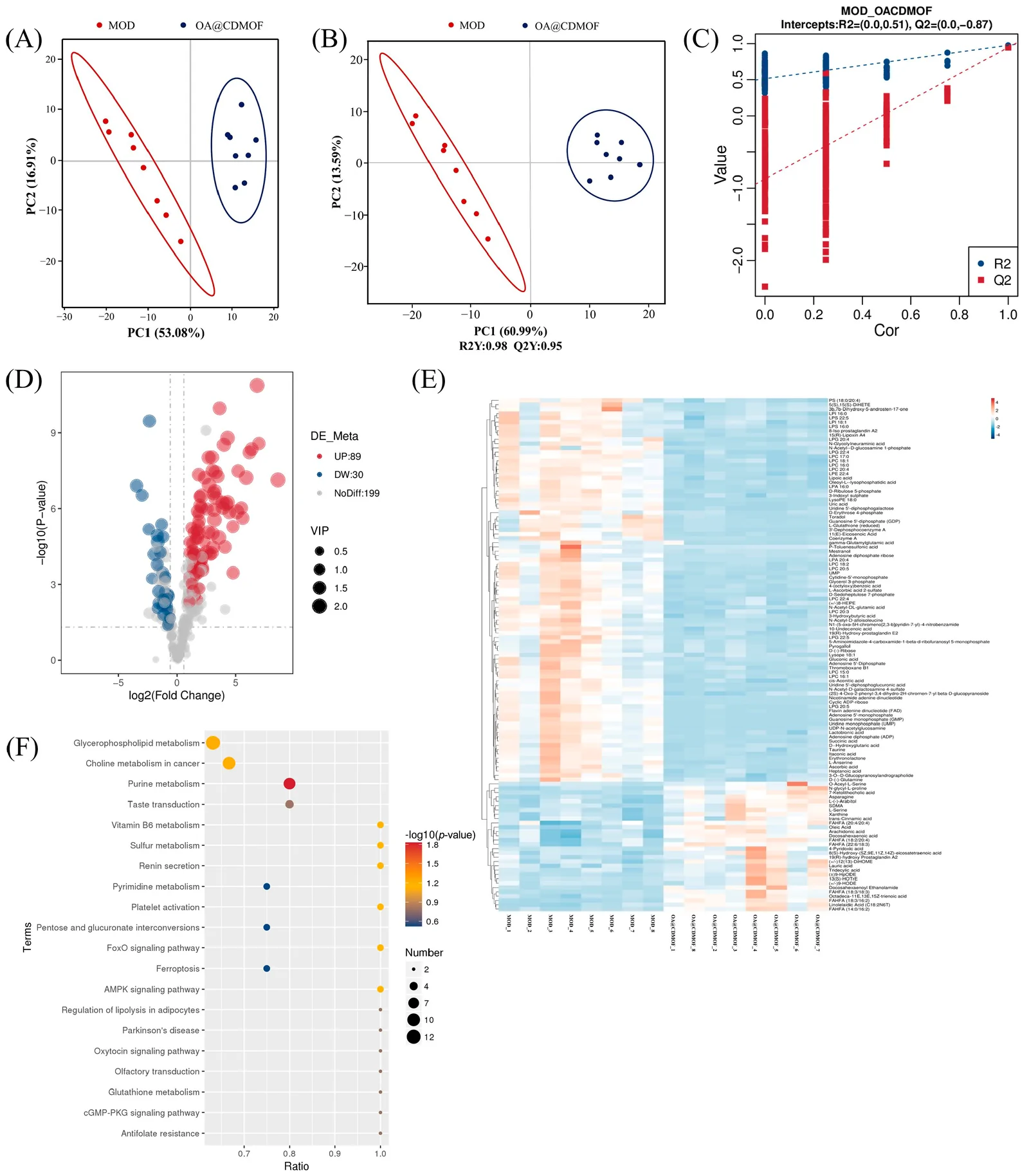
OA@CDMOF influences hepatic metabolites. (**A**) PCA and (**B**) OPLS-DA plots of liver metabolites. (**C**) Permutation test plot based on OPLS-DA. The blue and red dashed lines represent the linear regression fitting lines for R2 and Q2, respectively. (**D**) Volcano plot showing changes in the metabolites of the OA@CDMOF group relative to those of the MOD group. Heatmap of metabolites in the MOD and OA@CDMOF groups (**E**). OA@CDMOF influences metabolic pathways in HFD-induced mice (**F**). Only the MOD and OA@CDMOF groups were included in the metabolomics analysis. *n* = 8 mice per group.

To further assess whether OA@CDMOF resulted in metabolic alterations, liver samples were subjected to nontargeted metabolomic analysis. An HFD altered liver metabolites in normal mice, indicating that the NAFLD model was successfully constructed (Figure S1). To further assess the impact of OA@CDMOF on liver function, we also analyzed metabolites in the MOD and OA@CDMOF groups. The results from PCA and OPLS-DA revealed distinct differences in liver metabolites between mice in the MOD group and those treated with OA@CDMOF (Figure 4A,B). Subsequent permutation tests confirmed the reliability of the OPLS-DA models without overfitting (Figure 4C). Furthermore, a volcano plot (Figure 4D) highlights the significant alterations in liver metabolites following OA@CDMOF treatment. Specifically, 89 metabolites increased, while 30 metabolites decreased after OA@CDMOF intervention.

In the MOD group, many metabolites, including lipids and lipid-like molecules such as lysophosphatidylcholine (LPC 20:4) and fatty acid esters of hydroxy fatty acids (FAHFA 18:3/18:3), were markedly enriched in the OA@CDMOF-treated group. These findings suggest that OA@CDMOF may influence lipid metabolism and other critical pathways in the liver.

To further identify the key metabolites regulated by OA@CDMOF, a combination of OPLS-DA and a *t* test analysis was performed (VIP > 1 and *p* < 0.05), as shown in Figure 4. In the MOD group, many metabolites were enriched in the OA@CDMOF group, including lipids and lipid-like molecules (LPC 20:4, FAHFA (18:3/18:3), FAHFA (18:3/16:2), LysoPE 18:0, LPG 20:4, 11(E)-eicosenoic acid, LPG 20:5); nucleosides, nucleotides, and analogs (uridine monophosphate (UMP)), adenosine diphosphate ribose, adenosine diphosphate (ADP), nicotinamide adenine dinucleotide, adenosine 5′-diphosphate, Adenosine 5′-monophosphate, and uridine 5′-diphosphoglucuronic acid); organic oxygen compounds (cyclic ADP-ribose, d-ribulose 5-phosphate); and organoheterocyclic compounds (uric acid, erythronolaactone, and L-ascorbic acid 2-sulfate), which are involved in fatty acid and nucleotide metabolism, were markedly enriched. On the other hand, a few compounds in the OA@CDMOF group, including amino acid metabolism-related metabolites (such as N-glycyl-L-proline, L-serine, trans-cinnamic acid, oleic acid, arachidonic acid, docosahexaenoic acid, lauric acid, tridecylic acid, and linolelaidic acid), were detected (Figure 4E).

### 2.7. Effects of OA@CDMOF on Metabolic Pathways

The hepatic metabolite abundance differed between the MOD and OA@CDMOF groups, as reflected by PCA. To explore the metabolic pathways influenced by OA@CDMOF, differentially abundant metabolites were analyzed (VIP > 1 and *p* < 0.05) (Figure 4F). Overall, the results revealed that OA@CDMOF was associated with changes in metabolites related to glycerophospholipid, purine, glutathione, and vitamin B6 metabolism, as well as pentose and glucuronate interconversions; lipolysis regulation in adipocytes; and FoxO, AMPK, and cGMP-PKG signaling, among others. The identification of metabolites associated with AMPK, FoxO, and cGMP-PKG pathways is based on pathway enrichment analysis of untargeted metabolomics data. These associations are correlated and do not demonstrate direct activation of these signaling pathways. Confirmation would require targeted metabolite quantification and/or protein expression and phosphorylation analyses, which were not performed in this study. In particular, vitamin B6 emerged as a key target for the ability of OA@CDMOF to mitigate NAFLD. In this context, studies have reported that vitamin B6 levels are lower in NAFLD patients, with deficiency also considered a significant risk factor for disease [36]. Moreover, vitamin B6 has been shown to reduce hepatic fat deposition in HFD-induced Apoe−/− mice, potentially through its influence on homocysteine levels [37].

### 2.8. Effects of OA@CDMOF on the Gut Bacterial Community in Mice

**Figure 5:**
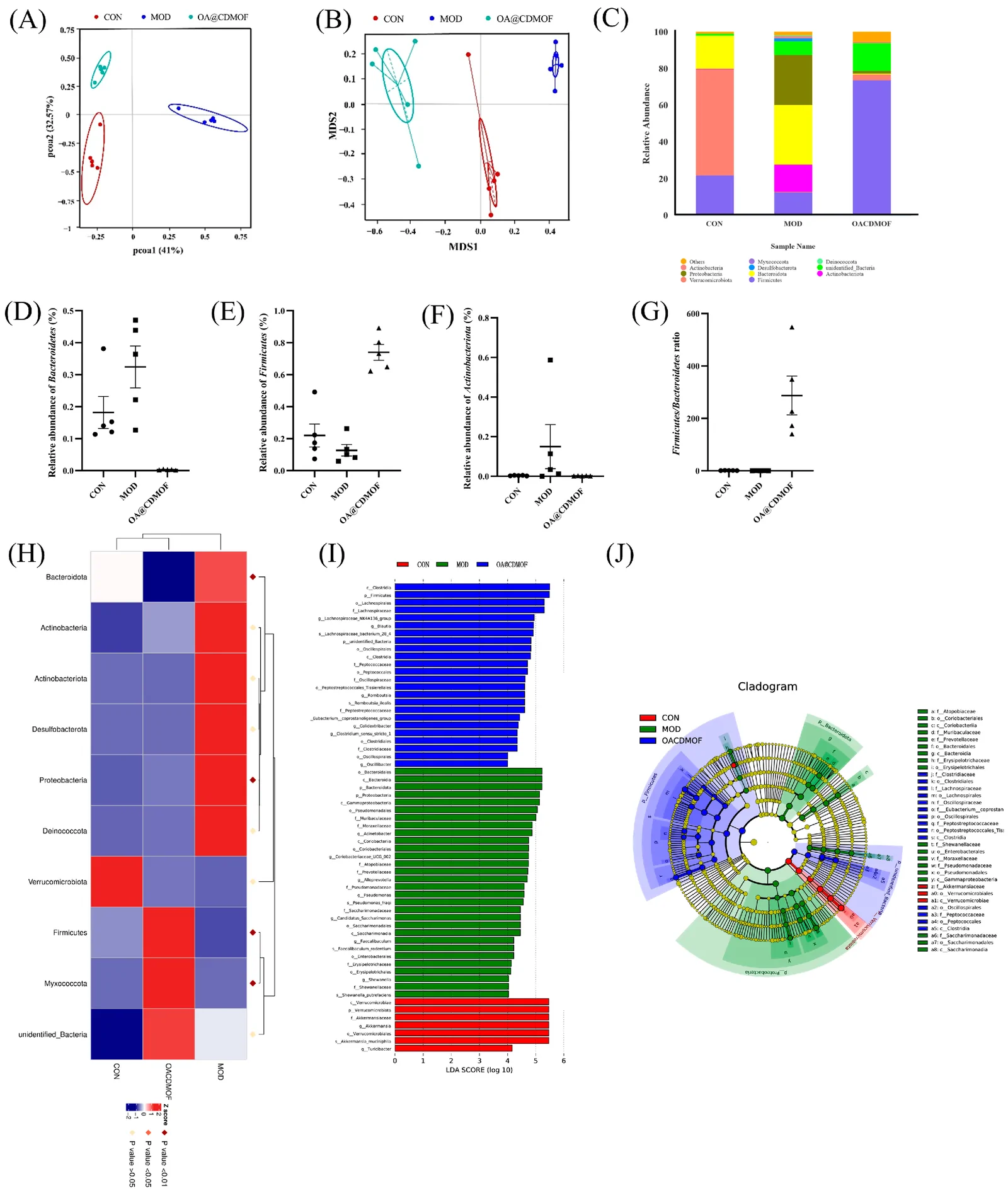
Effects of OA@CDMOF on the composition of gut microorganisms. (**A**) PCoA of the gut microbiota. (**B**) NMDS plots. (**C**) Top 10 most abundant bacterial phyla: *Bacteroidota* (**D**), *Firmicutes* (**E**), *Actinobacteriota* (**F**), and the *Firmicutes*/*Bacteroidota* ratio (**G**). (**H**) Heatmap of the top 10 most abundant bacterial phyla. Characteristic genera shown by LEfSe analysis (**I**). Distribution histogram of the LDA scores, with higher values indicating greater importance of the bacteria. (**J**) Cladograms: brown dots represent unimportant bacteria in all groups, and other colored dots are important bacteria in groups with the corresponding colors. Only the CON, MOD, and OA@CDMOF groups were included in the gut microbiota analysis; the free OA group was not included. The data are expressed as the mean ± SEM (*n* = 5). *p* < 0.05.

The gut microbiota is closely linked to NAFLD pathogenesis through the modulation of lipid metabolism, immune homeostasis and inflammation. To understand how OA@CDMOF influences gut microbes in NAFLD mice, 16S rRNA amplicon sequencing was conducted to analyze the overall microbial structure. In this case, PCoA revealed marked differences in the composition of the microbiota among the groups (Figure 5A), indicating that OA@CDMOF supplementation led to notable shifts in the microbial profiles. These changes were further supported by the NMDS analysis (Figure 5B). The microbiota was dominated by the phyla *Bacteroidota*, *Firmicutes*, and *Actinobacteriota* (Figure 5C). Specifically, mice in the MOD group presented increased abundances of *Bacteroidota*, *Proteobacteria*, and Actinobacteriota, whereas the abundances of *Firmicutes* and *Verrucomicrobiota* decreased, leading to a decreased *Firmicutes*/*Bacteroidota* (F/B) ratio. In contrast, OA@CDMOF treatment led to marked *Firmicutes* enrichment and very low abundance of *Bacteroidota*, resulting in a strikingly high F/B ratio (Figure 5D–G).

Analysis of the sequencing data indicated that specific gut bacterial species appeared to benefit from OA@CDMOF treatment and that these could alleviate NAFLD. An overview of the gut bacterial profile at the phylum level is shown in Figure 5H. The *Firmicutes*/*Bacteroidota* (F/B) ratio is considered a key indicator of intestinal homeostasis, with an elevated F/B ratio and an increase in the abundance of Actinobacteriota linked to obesity and metabolic imbalances, including NAFLD. Correlation analyses revealed that *Firmicutes* and *Actinobacteriota* were positively and significantly linked to body weight, serum lipids, liver damage, and inflammation in NAFLD mice, whereas Bacteroides was negatively associated with these parameters. Furthermore, in the MOD group, the abundance of bacteria significantly increased and the F/B ratio decreased, whereas OA@CDMOF treatment reversed these effects. The pattern of increased *Bacteroidota* and decreased *Firmicutes* with a lower F/B ratio observed in our MOD group is consistent with the findings of several published studies. A comprehensive review of 16S rRNA sequencing studies revealed that the majority of NAFLD/NASH patients exhibit increased Bacteroidota and a decreased F/B ratio [38]. A study in Thai NASH patients also revealed a significantly elevated Bacteroidota/Firmicutes ratio [39]. Furthermore, studies in Chinese MAFLD patients have reported increased abundance of Bacteroidota and Proteobacteria and decreased abundance of Firmicutes [40]. The increased abundance of Proteobacteria and reduced abundance of Verrucomicrobiota in our MOD group are also consistent with findings in NAFLD models [41] and with the decreased abundance of Akkermansia muciniphila, a key member of Verrucomicrobiota, observed in obese and type 2 diabetic mice [42].

*Firmicutes* and *Bacteroidota* together constitute more than 90% of the bacteria in the human intestine, and dysbiosis is linked to obesity. Changes in the F/B ratio are strongly linked to body weight, with higher ratios associated with obesity. Multiple studies have demonstrated that an increased Firmicutes to Bacteroidota ratio is closely associated with obesity, while phytochemicals have been shown to restore the balance between these two bacterial groups [43,44]. For instance, mice fed an HFD supplemented with 30 mg/kg/day 1,2,3,6-tetra-O-galloyl-β-D-glucopyranoside (GA) for 14 weeks experienced significant weight loss, which was accompanied by an increased proportion of *Bacteroidota* and a decrease in *Firmicutes* [45]. Notable differences were observed in the gut microbial profiles across treatment groups; in the MOD group, the F/B ratio was decreased, whereas OA@CDMOF treatment significantly elevated the F/B ratio by enriching Firmicutes and reducing Bacteroidota (Figure 5D–G). These changes suggest that rebalancing of the gut microbiota, rather than the absolute direction of the F/B ratio, may be a potential mechanism through which OA@CDMOF mitigates NAFLD.

Concurrently, a heatmap was constructed to show the relative abundances of the top 10 most abundant bacterial phyla (Figure 5H), and several phyla in the OA@CDMOF group were altered in the same direction as those in the CON group relative to those in the MOD group (Figure 5H). Specifically, the relative abundances of *Bacteroidota*, *Actinobacteriota*, *Desulfobacterota*, *Proteobacteria*, and *Deinococcota* decreased in the OA@CDMOF group, whereas those of *Firmicutes*, *Myxococcota*, and unidentified bacteria increased (Figure 5H). The characteristic bacteria in each group were then identified by LEfSe (Figure 5I). The findings revealed a total of 61 different bacteria across the three groups, of which 24, 30 and 7 were significantly different in the CON, MOD and OA@CDMOF groups, respectively. In particular, MOD intervention increased the relative abundance of *o_Bacteroidales*, *c_Bacteroidia*, *p_Bacteroidota*, *p_Proteobacteria*, *c_Gammaproteobacteria*, o_*Pseudomonadales*, and f_*Muribaculaceae*. The abundance of *c_Clostridia*, *p_Firmicutes*, *o_Lachnospirales*, *f_Lachnospiraceae*, *g_Lachnospiraceae NK4A136_group*, *g_Blautia*, *s_Lachnospiraceae bacterium_28_4*, *p_unidentified Bacteria*, *o_Oscillospirales*, *c_Clostridia*, *f_Peptococcaceae*, *o_Peptococcales*, *f_Oscillospiraceae*, *o_Peptostreptococcales Tissierellales*, *g_Romboutsia*, *s_Romboutsia ilealis*, *f_Peptostreptococcaceae*, *Eubacterium coprostanoligenes_group*, *g_Colidextribacter*, *g_Clostridium sensu stricto_1*, *o_Clostridiales*, *f_Clostridiaceae*, *o_Oscillospirales*, and *g*_*Oscillibacter* increased in the OA@CDMOF group (Figure 5I). For each treatment group, the key bacteria identified by LEfSe were further confirmed by a cladogram analysis (Figure 5J). These results were used to determine differences in the key microbiota enriched by OA@CDMOF. The findings indicated that OA@CDMOF treatment significantly increased the relative abundance of *f_Clostridiaceaek*, *o_Clostridiales*, *f_Lachnospiraceae*, *o_Lachnospirales*, *f_Oscillospiraceae*, *f_Eubacterium_coprostan*, *o_Oscillospirales*, *f_Peptostreptococcaceae*, *o_Peptostreptococcales*, *c_Clostridia*, *o_Oscillospirales*, *f_Peptococcaceae*, *o_Peptococcales*, and *c_Clostridia* (Figure 5J).

Interestingly, OA@CDMOF treatment markedly increased the abundance of *Verrucomicrobiota*, which is known to be linked to weight regulation. Phytochemicals influence their growth, with apple polyphenols reducing body weight and increasing the proportion of *Verrucomicrobiota* in C57BL/6J mice [46]. Consistently, the increased abundance of *Verrucomicrobiota* observed in this study suggests that OA@CDMOF may act as a potential prebiotic, promoting the growth of this beneficial microbiota.

In addition to *Verrucomicrobiota*, OA@CDMOF intervention also increased the abundances of *Oscillospirales*, [Eubacterium]_*coprostanoligenes*_group, *Prevotellaceae*, c_*Clostridia*, p_*Firmicutes*, o_*Lachnospirales*, and f_*Lachnospiraceae*. Several of these microorganisms, including *Oscillospirales* and *Prevotellaceae*, produce SCFAs, thus promoting weight control and preventing NAFLD. Therefore, the anti-NAFLD effects of OA@CDMOF could be partially mediated through its influence on the levels of *Oscillospirales* and *Prevotellaceae*.

Additionally, the [Eubacterium]_*coprostanoligenes*_group can mitigate HFD-induced dyslipidemia through the modulation of sphingosine metabolism [47], whereas the abundance of *Bacteroides*_sartorii is increased by theabrownin to mitigate metabolic syndrome. These findings indicate that OA@CDMOF enrichment of gut microbes contributes to reducing obesity and protecting the liver.

### 2.9. Limitations

Notably, a physical mixture of OA and CDMOF was not included as a control in the characterization experiments. This represents a limitation of the current study, as the control would have helped distinguish between true encapsulation and simple physical mixing effects. Future studies should include such a control to strengthen the characterization conclusions.

A major limitation of the present study is the absence of a CDMOF-only (carrier control) group. γ-CD and the potassium-containing framework may independently influence lipid metabolism, body weight, and the gut microbial composition. Without a carrier control, we cannot definitively attribute the observed therapeutic effects specifically to enhanced OA delivery. Therefore, the effects of OA@CDMOF reported here should be interpreted as the overall effects of the formulation, and a contribution from the carrier cannot be excluded. The absence of a statistically significant difference between OA and OA@CDMOF further suggests that the carrier may contribute to the observed effects. This limitation should be addressed in future studies by including a CDMOF-only group to isolate the effects of the carrier from those of the encapsulated OA.

Notably, the gut microbiota and liver metabolomics analyses compared only the MOD and OA@CDMOF groups and did not include the free OA group. Consequently, we cannot determine whether the observed changes in microbiota and metabolites are unique to the OA@CDMOF formulation or would also occur with free OA administration. Therefore, the effects of OA@CDMOF on the gut microbiota and liver metabolome should be interpreted as the overall effects of the OA@CDMOF formulation, and no conclusion can be drawn regarding whether OA@CDMOF differs from free OA in these omics analyses. This is an important limitation that should be addressed in future studies by including all treatment groups (CON, MOD, OA, OA@CDMOF) in multiomics analyses.

While Spearman correlation analysis revealed associations between specific microbial genera and liver metabolites, a true cross-omics integration linking microbial taxa, functional gene annotations, hepatic metabolic pathways, and phenotypic endpoints was not performed. Advanced multiomics integration methods (e.g., multiomics factor analysis and network-based integration) should be applied in future studies to better elucidate the gut–liver axis mechanisms.

## 3. Materials and Methods

### 3.1. Materials and Chemicals

OA (97%) and γ-CD (98% purity) were purchased from Macklin Reagent (Shanghai, China), while potassium hydroxide (KOH), AR-grade ethanol and methanol (MeOH) were obtained from Sinopharm Chemical Reagent Co., Ltd. (SCRC, Shanghai, China). In addition, TNF-α and IL-1β were obtained from Beyotime Biotechnology (Zhengzhou, China), while kits for testing LDL-c, HDL-c, triglyceride (TG) and total cholesterol (TC) levels as well as serum levels of SOD, ALT, MDA, and AST were obtained from Nanjing Jiancheng Bioengineering Institute (Nanjing, China).

### 3.2. Preparation of CDMOF and OA@CDMOF

CDMOF was prepared as previously reported by the current group [48]. Briefly, 8.0 mmol of KOH (0.45 g) and 1.0 mmol of γ-CD (approximately 1.32 g) were placed in 40 mL of pure water, and after 4 h of continuous mixing on a magnetic stirrer at 25 °C, the mixture was kept in a vacuum desiccator at 25 °C for 14 days, after which the mixture was sealed with 50 mL of methanol, and it was allowed to undergo vapor diffusion. This was followed by centrifugation, after which the resulting CDMOF crystals were rinsed with three changes of 15 mL of anhydrous ethanol for the removal of unreacted reagents. Finally, the crystals were dried in an oven for 24 h at 60 °C to yield a white powder composed of cubic crystals. The powder was preserved under cool, dry, airproof, and pollution-free conditions.

The mass ratio of OA to CDMOF (1:10, *w*/*w*) was selected on the basis of preliminary optimization. OA has extremely low aqueous solubility (approximately 4.31 µg/L), which limits its ability to be effectively encapsulated into CDMOF. High OA loading ratios resulted in poor encapsulation efficiency with significant amounts of unencapsulated OA, whereas low ratios resulted in insufficient drug content. The 1:10 ratio provided an optimal balance between drug loading and encapsulation efficiency.

OA (0.03 g) and CDMOF (0.3 g) were added to 30 mL of water, and after 30 min of continuous stirring at 50 °C, the resulting heterogeneous solution was vacuum filtered to obtain a clear liquid. This was followed by vacuum freeze-drying, which was performed overnight until a yellow powder of OA@CDMOF was obtained.

### 3.3. OA Loading and Solubility Analysis of OA@CDMOF

#### 3.3.1. HPLC Analysis

The OA concentration of the samples was assessed by HPLC (LC-20A, Shimadzu, Kyoto, Japan) with a C18 column (4.6 × 150 mm, 5 μm; Thermo Fisher Scientific, Waltham, MA, USA) at room temperature [15]. The separation was performed at a flow rate of 1.0 mL/min, with methanol-water-phosphoric acid (90:10:0.1, *v*/*v*/*v*) used as the mobile phase. Detection was eventually achieved at 210 nm with a UV detector. The calibration curve for OA was constructed over the range of 0.01–10 mg/L (10–10,000 ng/mL), with a correlation coefficient (R^2^) > 0.999. The limit of detection (LOD) and limit of quantification (LOQ) were 0.005 mg/L and 0.01 mg/L, respectively.

#### 3.3.2. Encapsulation Efficiency (EE) and Solubility

For OA loading analysis, OA@CDMOF (20 mg) was dispersed in distilled water (100 mL) and shaken at 37 °C for 24 h. After centrifugation (4500 rpm, 20 min), the amount of OA in the supernatant was assessed by HPLC (λ = 210 nm). This method measures the amount of OA that can be released into aqueous solution under the tested conditions rather than the total OA incorporated. The EE calculation is based on the amount of OA recovered in the aqueous phase relative to the total OA added during preparation, and this may underestimate the true encapsulation if some OA remains trapped within the CDMOF structure. On the basis of the results, the encapsulation efficiency (EE) was determined as follows:

(1) $$\mathrm{EE}=\frac{\mathrm{Amount of encapsulated OA}}{\mathrm{Total amount of OA}}\times 100\%$$

To assess the solubility, excess OA@CDMOF (approximately 50 mg) was added to 10 mL of distilled water (pH 6.8) in a sealed vial. The mixture was shaken at 37 °C and 150 rpm for 48 h to ensure equilibrium, which was confirmed by measuring the OA concentration at 24 h and 48 h (no significant difference was observed). After equilibration, the suspension was centrifuged at 15,000 rpm for 10 min, and the supernatant was filtered through a 0.45 μm polyethersulfone (PES) membrane filter. The filtrate was then mixed with the mobile phase prior to HPLC analysis. All the solubility measurements were performed in triplicate (*n* = 3).

### 3.4. Characterization

The chemical composition of the samples was qualitatively analyzed by FTIR (Thermo Nicolet iS5, Waltham, MA, USA). In this case, powdered samples were mixed with KBr and pressed into transparent flakes, which were then kept on a sample holder for scanning over 400–4000 cm^−1^. In addition, the crystal structures of the samples were analyzed by XRD at room temperature (Bruker D8 Advance diffractometer, Karlsruhe, Germany; CuKα radiation (λ = 1.54 Å), 40 kV, and 40 mA), with measurements taken at a 2θ diffraction angle range of 5–40 degrees. Thermal stability was assessed with a thermal analysis system (Shimadzu DTG-60). The sample (approximately 5 mg) was warmed at a rate of 10 °C min^−1^ under nitrogen until it reached 600 °C. Nitrogen adsorption–desorption isotherms were measured with a porosimeter (Micromeritics ASAP 2460, Norcross, GA, USA), with surface area determined using the Brunauer–Emmett–Teller (BET) method. Morphological characterization of CDMOF and OA@CDMOF was performed via SU3500 SEM (S-3400 N; Carl Zeiss, Oberkochen, Germany). The samples were distributed on conductive adhesive tape, followed by gold plating.

### 3.5. Animals

#### 3.5.1. Experimental Design for Animal Grouping and Treatment

Thirty-two male C57BL/6J mice (6–8 weeks old, 19–21 g) were obtained from Jiangsu Ailingfei Biotechnology Co., Ltd. (Nanjing, China). The mice were allowed to acclimatize for seven days with freely available food and water at 23 ± 2 °C and 50–60% humidity along with a 12 h light/dark cycle. Animal experiments were conducted in accordance with the National Research Council’s Guide for the Care and Use of Laboratory Animals, and the experimental protocols were conducted in accordance with the ARRIVE guidelines and approved by the Experimental Animal Welfare Ethics Review Committee of Jiangsu Ailingfei Biotechnology Co., Ltd. (License No.: JSAB24014M), Nanjing, China.

The mice were fed either a low-fat diet (CON, control group) or a high-fat diet (MOD, model group) (Jiangsu Xietong Pharmaceutical Bioengineering Co., Ltd., Nanjing, China). After eight weeks, the mice were divided into four groups: (1) low-fat diet (CON, *n* = 8); (2) high-fat diet model (MOD, *n* = 8); (3) MOD with free OA (OA, 5 mg/kg/day, equivalent to 0.1 mg OA per 20 g mouse); and (4) MOD with OA@CDMOF (OA@CDMOF, 5 mg/kg/day OA equivalent, based on the measured OA content of 11.38 mg/g, corresponding to approximately 8.8 mg OA@CDMOF powder per 20 g mouse) administered by gavage for four weeks. Given the extremely low aqueous solubility of OA, both free OA and OA@CDMOF were administered as aqueous suspensions rather than fully dissolved solutions; the suspensions were prepared with 0.5% CMC-Na as the suspending agent, and each mouse received a fixed dosing volume of 0.2 mL per 20 g body weight (10 mL/kg). For OA@CDMOF, this corresponded to a suspension concentration of approximately 44 mg/mL, delivering the OA-equivalent doses described above. Body weights were measured weekly, and at completion of the treatment, mice were anesthetized and blood taken from the retroorbital sinus. Livers and fat tissues were harvested, weighed, and kept at −80 °C.

#### 3.5.2. Biochemical Analyses

Serum levels of TG, TC, AST, ALT, HDL-c, and LDL-c were measured using a fully automated biochemical analyzer (Chemray 800, Shenzhen, China). Liver concentrations of TC, TG, MDA, and SOD were determined using commercial kits (Nanjing Jiancheng Bioengineering Institute, China; catalog numbers: A111-1 for TC, A110-1 for TG, A003-1 for MDA, A001-3 for SOD). SOD activity was determined by the xanthine oxidase method (WST-1 assay), and MDA activity was measured by the thiobarbituric acid (TBA) method. TNF-α and IL-1β levels in liver homogenates were measured using ELISA kits (Beyotime Biotechnology, Zhengzhou, China; catalog numbers: PI303 for TNF-α and PI301 for IL-1β). All the assays were performed according to the manufacturer’s instructions. Cytokine levels were normalized to total protein content determined by the BCA method.

#### 3.5.3. Histopathological Evaluations

After the mouse tissues were fixed in 4% formalin, the tissue sections (4 μm thick) were stained with hematoxylin and eosin (H&E) for histopathological analyses. Images were acquired under a light microscope at 200× magnification (Olympus, Tokyo, Japan).

#### 3.5.4. Untargeted Metabolomics in the Liver

The tissues were ground in liquid nitrogen and then mixed thoroughly with cold 80% methanol and 0.1% formic acid. Equal amounts of extracts were combined to form a QC sample, with blank samples also included to account for background ions. For the relative quantification of metabolites, a Vanquish UHPLC system (Thermo Fisher Scientific, Waltham, MA, USA) coupled with an Orbitrap Q Exactive series mass spectrometer (Thermo Fisher) was used. Both positive and negative ion modes were employed for untargeted metabolite detection, with the metabolites subsequently identified and quantified by comparison of retention times and mass-to-charge ratios (*m*/*z*) against reference databases. The metabolome data were eventually obtained after the removal of background ions and subsequent data normalization.

To assess differences in liver metabolites across groups, principal component analysis (PCA) and orthogonal partial least squares discriminant analysis (OPLS-DA) were conducted using SIMCA-P 16.0. The variable importance projection (VIP) value was then used to identify metabolic indicators, with *p* < 0.05 and a VIP > 1 used. Metabolite-associated pathways were examined using the KEGG database.

#### 3.5.5. Gut Microbiota Analysis

Sequencing was conducted as previously described. Total genomic DNA was extracted from mouse fecal samples, and the V3–V4 hypervariable region of the 16S rRNA gene was amplified by PCR using specific primers. The PCR products were subsequently purified, and paired-end sequencing was performed on the Illumina NovaSeq platform. Sequence data were processed using the QIIME2 pipeline, denoised with DADA2, and taxonomically annotated against the SILVA database. Differential taxa among groups were identified using linear discriminant analysis effect size (LEfSe) analysis. Sequencing was performed by Novogene Co., Ltd. (Beijing, China).

### 3.6. Statistical Analyses

The results are presented as the means ± SEMs. To assess the significance of differences, one-way ANOVA, followed by Tukey’s post hoc test, was conducted with GraphPad Prism software (version 10.4.1), with *p* < 0.05 considered to indicate statistical significance. Finally, Spearman correlation analysis was performed to determine potential correlations between the gut microbiota and liver metabolites.

## 4. Conclusions

In conclusion, we confirmed that OA@CDMOF can protect against NAFLD, with effects comparable to those of free OA, with no statistically significant difference between the two groups. The results of the present study revealed that OA@CDMOF could alleviate NAFLD in mice through combined actions, such as reducing the levels of adipokines and inflammatory mediators, increasing liver antioxidant capacity, decreasing blood lipid deposition and reducing liver fat accumulation. Combining metabolomics and 16S rRNA sequencing revealed that OA@CDMOF was associated with changes in metabolites related to glycerophospholipid, purine, glutathione, and vitamin B6 metabolism; pentose and glucuronate interconversions; lipolysis regulation in adipocytes; and FoxO, AMPK, and cGMP-PKG signaling.

However, while current findings from animal models provide valuable insight into the therapeutic potential of OA@CDMOF for NAFLD, it is important to recognize that these models do not fully replicate the complexity of human disease. Therefore, although these results offer a strong scientific foundation, further clinical studies are essential to validate the therapeutic efficacy of OA@CDMOF in humans and to explore its full potential as a treatment for NAFLD.

## Data Availability

Data are contained within the article and Supplementary Materials.

## Supplementary Materials

The following supporting information can be downloaded at https://www.mdpi.com/article/10.3390/molecules31203663/s1, Figure S1: Effects of an HFD on liver metabolites. (A) PCA and (B) OPLS-DA plots of liver metabolites. (C) Permutation test based on OPLS-DA. (D) Volcano plot of changes in metabolites between the CON and MOD groups. (E) Heatmap of the metabolites in the MOD and CON groups. (F) HFD consumption regulates metabolic pathways in mice. *n* = 8 mice per group.
