# Supplementary material for: Oleanolic Acid-Loaded Cyclodextrin Metal–Organic Framework Ameliorates NAFLD via Modulation of Gut Microbiota and Liver Metabolism
Source: Molecules. 2026 Oct 13;31(20):3663. doi: 10.3390/molecules31203663 (PMC13707472; doi:10.3390/molecules31203663)
Supplement: Supplementary file 1 [file molecules-31-03663-s001.zip › molecules-4565855-supplementary-REVISED.pdf]

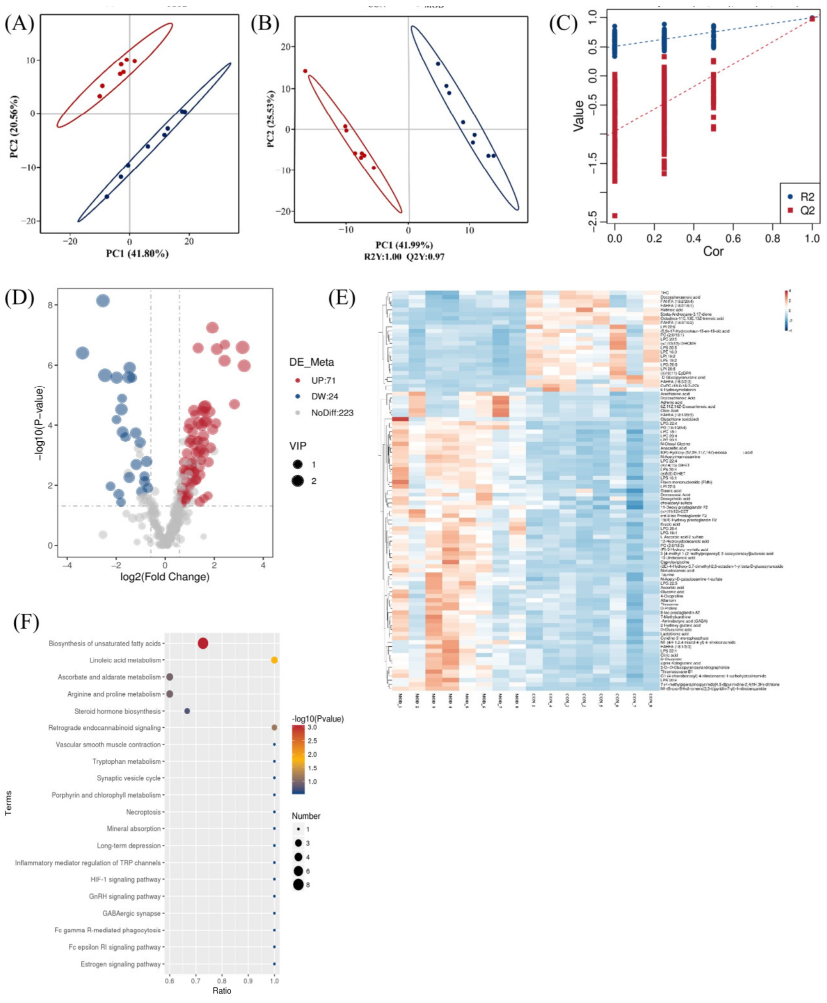

Figure. S1. Effects of HFD on liver metabolites. (**A**) PCA and (**B**)OPLS-DA plots of liver metabolites. (**C**) Permutation test based on OPLS-DA. (**D**) Volcano plot of changes in metabolites between the CON and MOD groups. (**E**) Heatmap of the metabolites in the MOD and CON groups. (**F**) HFD regulates the metabolic pathways in mice. $n = 8$/mice per group.
